# A tool for safer prescribing in vulnerable adults: the continuing development of the Medichec app and website

**DOI:** 10.1192/bjb.2023.71

**Published:** 2024-08

**Authors:** Delia Bishara, Sahar Riaz, Justin Sauer, Christoph Mueller, Siobhan Gee, David Taylor, Robyn-Jenia Wilcha, Millie Edwards, Nirja Beehuspoteea, Anne Marie Bonnici Mallia, Jennifer Brook, Bharathi Balasundaram, Daniel Harwood, Nicola Funnell, Andre Strydom, Robert Stewart

**Affiliations:** 1 Mental Health of Older Adults and Dementia Department, South London and Maudsley NHS Foundation Trust, London, UK; 2 Institute of Psychiatry, Psychology & Neuroscience, King's College London, London, UK; 3 Pharmacy Department, South London and Maudsley NHS Foundation Trust, London, UK; 4 Changi General Hospital, Singapore

**Keywords:** Medichec, side-effects, anticholinergic, dementia, medication

## Abstract

**Aims and method:**

Adverse effects are a common concern when prescribing and reviewing medication, particularly in vulnerable adults such as older people and those with intellectual disability. This paper describes the development of an app giving information on side-effects, called Medichec, and provides a description of the processes involved in its development and how drugs were rated for each side-effect. Medications with central anticholinergic action, dizziness, drowsiness, hyponatraemia, QTc prolongation, bleeding and constipation were identified using the British National Formulary (BNF) and frequency of occurrence of these effects was determined using the BNF, product information and electronic searches, including PubMed.

**Results:**

Medications were rated using a traffic light system according to how commonly the adverse effect was known to occur or the severity of the effect.

**Clinical implications:**

Medichec can facilitate access to side-effects information for multiple medications, aid clinical decision-making, optimise treatment and improve patient safety in vulnerable adults.

It is widely recognised that people with dementia^1,2^ and intellectual disability^3^ have increased vulnerability to adverse events from medication, and yet are also more likely to be receiving polypharmacy because of high rates of multimorbidity. Dementia itself is already associated with frailty,^4–6^ orthostatic hypotension^7,8^ and falls.^8^ In addition, people with cognitive impairment or intellectual disability are less likely to report medication side-effects, many of which may be asymptomatic or difficult to detect, until they become potentially life threatening.

Medichec began as a means of communicating information on medications with central anticholinergic actions. Medications with anticholinergic activity are frequently associated with adverse effects and poor outcomes in vulnerable adults with physical comorbidities, such as older people and those with intellectual disability. As well as the well-recognised peripheral anticholinergic side-effects (e.g. dry mouth, blurred vision, constipation and urinary retention) anticholinergic drugs can also cause central adverse effects (e.g. drowsiness, delirium and cognitive impairment).^9,10^ Furthermore, in the past 15 years, there has been a considerable amount of data emerging suggesting that these agents can increase the risk for dementia and mortality in older people.^11,12^

The Anticholinergic Cognitive Burden (ACB) scale had been widely used to identify drugs with high anticholinergic burden for patients,^13^ but the degree of penetration of drugs across the blood–brain barrier and their receptor selectivity do not appear to have been considered in the functionality of this scale. This is evident for the bladder anticholinergic drug group, in which all agents are allocated a score of 3 (the highest possible score) despite their physiochemical differences that allow some to penetrate the blood–brain barrier more than others. As a result, a team at the South London and Maudsley NHS Foundation Trust (SLaM) decided to develop a new central anticholinergic burden scale. The team took all the drugs’ relevant physiochemical factors into account and developed the Anticholinergic Effect on Cognition (AEC) scale, which specifically looked at the central anticholinergic action of drugs.^14^ The step-by-step development of the AEC scale has been reported elsewhere^14^ and the scale has been clinically validated in people with dementia.^15^ It has also been shown to have greatest predictive accuracy for dementia/delirium when compared with other scales.^16^

The implementation of the AEC scale in SLaM memory services was successful and greatly improved the identification of high-risk anticholinergic drugs and subsequent communication to general practitioners (GPs) regarding medication reviews.^17^ However, owing to the risk of human error arising from misidentification of drugs from the list or simple calculation errors when adding up total scores, the need for an electronic version of the AEC scale became quickly apparent. This led to the development of the first version of Medichec, a free website tool based on the AEC scale. It can also be downloaded to smartphones or tablets as an Apple or Android app for a small fee. This functionality, maintained in all subsequent versions, allows practitioners to easily check a patient's medication, and the cumulative anticholinergic burden score is calculated automatically. The system will identify a drug and its score by any of the names listed in the British National Formulary (BNF). It also alerts the practitioner as to which drugs need to be reviewed and gives advice on how to interpret the score. Medichec uses generic names and accesses over 2000 medications. Clinical staff can use the app and incorporate results into their reports, or they can communicate the scores directly to GPs, as results can also be emailed from the website.

The success and increasing use of Medichec to identify central anticholinergic burden highlighted the importance of having side-effect data easily available and accessible to clinicians. Further development of the app has subsequently been carried out with the addition of several adverse effects that are paramount to the safety of prescribing in vulnerable individuals, such as older people and those with intellectual disability. These include dizziness and drowsiness, made available in Version 2 of the site/app, and more recently QTc prolongation, hyponatraemia, bleeding risk and constipation.

This paper aims to explain the reasoning for the selection of adverse effects now incorporated into Medichec, to describe the processes involved in the development of the medication lists accessed by it and to define how drugs were rated for each adverse effect.

## Rationale for adverse effects, beyond central anticholinergic action, included in more recent versions of Medichec

### Dizziness

Dizziness as a symptom is highly prevalent among older people and is associated with many health factors, including falls, lower health-related quality of life, disability and depression/anxiety.^18^ Although dizziness can occur from other drug-related effects, such as hypoglycaemia or vestibular system dysfunction, orthostatic hypotension can also result in symptoms of dizziness,^19^ underscoring the need for early detection of potential causes and preventive strategies. Dizziness is reported as an adverse effect of many medications and may be a risk factor for adverse outcomes in those affected, regardless of whether or not there is demonstrable orthostatic hypotension as an underlying cause of the symptom. In addition, it can worsen cognitive impairment and confusion.^20^

### Drowsiness/sedation

Another important adverse effect is sedation or drowsiness, given the risk of oversedation and its impact on accelerated cognitive decline and hospital admissions in older people,^21^ in addition to increased falls and fractures.^22^ Most centrally acting anticholinergic medications cause sedation, but not all sedative medications are anticholinergic, and several other neurotransmitter systems are involved, including the noradrenergic, glutaminergic, histaminic, opioid and gamma-aminobutyric acid (GABA)-ergic system.^21^

### QTc prolongation

The electrocardiogram (ECG) QT interval measures the time for myocardial cells to depolarise and repolarise and is conventionally corrected for the heart rate, resulting in the measured QTc interval. Patients with a prolonged QTc interval are at risk of ventricular arrhythmias, torsades de pointes and sudden death.^23,24^ Electrolyte disturbances as well as cardiac conduction abnormalities such as heart block and bradycardia predispose to arrhythmias. People with an intellectual disability are more likely to have congenital heart conditions, and older people are more likely to be electrolyte deficient, especially if they are receiving diuretics, and are thus at increased risk for the effects of QTc prolongation. The QTc interval may also increase with age in normal hearts owing to physiological ageing processes in the myocardium.^25,26^ Concomitant usage of drugs prolonging the ECG QTc interval is common in older people and in those with intellectual disability and although substantial evidence does not yet exist, it is commonly assumed that the QTc interval will increase with the number of co-administered QTc-prolonging drugs.^25^

### Hyponatraemia

Hyponatraemia is a relatively common electrolyte abnormality seen in clinical practice and is especially prevalent in older people because age is a strong independent risk factor.^27,28^ Other contributing factors include the presence of factors contributing to increased antidiuretic hormone as well as the prescription of drugs associated with hyponatraemia (e.g. diuretics) and mechanisms such as the ‘tea and toast’ syndrome.^29^ It has been shown that concurrent use of hyponatraemia-inducing medications increased the risk of severe hyponatraemia compared with that with individually administered hyponatraemia-inducing medication.^30^

Hyponatraemia has serious implications and is associated with impaired balance, falls, hip fractures, osteoporosis and cognitive dysfunction.^31–34^ Even mild, apparently asymptomatic hyponatraemia is associated with prolonged stays in hospital.^35^ In addition, it is associated with increased mortality in older populations.^27,36^ Many individuals with intellectual disability have comorbid epilepsy, and several anti-epileptics may predispose to hyponatraemia.^37^

### Bleeding risk

Drug-induced bleeding presents in many ways in older people, including excessive bruising, nosebleeds, and gastrointestinal, rectal and intracranial bleeding. As with many other adverse effects, advanced age is a risk factor for medication-related bleeds.^38^ Drug-induced bleeding is potentiated by numerous drugs and drug–drug interactions.^39^ It is important to be aware of patient-specific risk factors but also to easily identify medications that contribute to bleeding. By minimising the risk of drug-induced bleeding in patients, clinicians and pharmacists have the opportunity to reduce long-term adverse effects**.**

### Constipation

The prevalence of constipation rises dramatically with age, with some estimates approaching 50% among people over 80 years old.^40^ Among people with intellectual disability, similar prevalence rates have been found.^41^ Constipation is often multifactorial and can be caused by certain disease states and conditions, such as anxiety, Parkinson's disease, hormonal changes or metabolic factors, medications such as opioids and anticholinergic medication, as well as lifestyle factors.^42^ Consequences of constipation can be serious and substantial and are often underestimated. In susceptible older people who are frail, excessive straining can trigger a syncopal episode, or coronary or cerebral ischaemia. Less acutely, constipation leading to faecal impaction can present with anorexia, nausea and pain associated with functional decline. Case reports have identified bowel ulceration, perforation and death as consequences of faecal impaction.^40,43^ Quality of life also appears to be lower for older people with constipation, it is one of the most frequent causes of behavioural and psychological symptoms of dementia (BPSD)^44^ and long-term care facilities often incur higher costs managing this problem.^40^ In addition, when laxatives are used to treat constipation, this can lead to falls.^42^

## Medichec expansion and development

As part of the continuing development of Medichec we searched the electronic BNF for each adverse effect within the drug treatment summaries to determine which drugs could cause the side-effects listed above. A measure of the frequency or likelihood of these adverse effects occurring was recorded. The BNF uses the World Health Organization (WHO) definitions and classifies frequencies of side-effects as follows:

- very common: greater than 1 in 10
- common: 1 in 100 to 1 in 10
- uncommon: 1 in 1000 to 1 in 100
- rare: 1 in 10 000 to 1 in 1000
- very rare: less than 1 in 10 000
- frequency unknown: either the frequency is not defined by product literature or the side-effect has been reported from post-marketing surveillance data.

### The traffic light rating system

Medications were classified using an extended traffic light system according to how commonly the adverse effect was known to occur or the severity of the effect.

### Dizziness

Drugs associated with ‘orthostatic hypotension’ and ‘dizziness’ were identified by searching for each side-effect in the BNF database hosted by the National Institute for Health and Care Excellence (NICE) (at bnf.nice.org.uk). From the identified results, only those drugs where the search term was listed as a side-effect with known frequency were accepted. Drugs where the search term was present as a caution or contraindication but not a side-effect, or where it was only listed as a side-effect in overdose, were excluded. This process was repeated using a second database, the BNF available through MedicinesComplete database (www.pharmaceuticalpress.com/products/british-national-formulary), to perform a separate search and verify the list of drugs identified by the first database. The same search criteria were used to compare up to the first 200 results for each separate search term. Once a list of medications causing dizziness and another list for orthostatic hypotension had been drawn up, it was seen that the overlap between the two lists consisted of certain additional drugs causing dizziness, but no additional drugs causing only orthostatic hypotension; therefore, the side-effect ‘dizziness’ was used for the Medichec database.

Drugs causing dizziness were classified in Medichec as follows:

- Dizziness not reported in the BNF/Frequency not known/Drug not analysed for this side-effect.
- Dizziness reported as uncommon, rare or very rare.
- Dizziness reported as common or very common.

### Drowsiness/sedation

For the drowsiness list, the NICE BNF database was used, and the following search words were entered: ‘drowsiness’, ‘somnolence’ or ‘sedation’ listed as a side-effect in drug treatment summaries. A list of drugs and frequencies was compiled in the same way as for dizziness.

Drugs causing drowsiness were classified in Medichec as follows:

- Drowsiness not reported in the BNF/frequency not known/drug not analysed for this side-effect.
- Drowsiness reported as uncommon, rare or very rare.
- Drowsiness reported as common or very common.
- Drowsiness reported as common or very common AND drug has an additional cautionary warning for drowsiness in the BNF. Within this group are some antipsychotic drugs deemed particularly sedating (as per Maudsley Prescribing Guidelines^45^) and anticholinergic drugs that are centrally acting.

### QTc prolongation

The NICE BNF website was used to identify drugs listed as having QTc prolongation anywhere in the drug treatment summary. The search term ‘QT’ was used. This included cautions, contraindications, drug interactions and side-effects (because of the severity of this adverse effect, it was felt important also to identify drugs where QTc is listed as a caution, contraindication or drug interaction). If the BNF listed the frequency as not known, the drug's Summary of Product Characteristics (SPC) was checked, using the electronic Medicines Compendium (emc) website (www.medicines.org.uk/emc). Finally, to confirm and supplement the findings, the electronic resource CredibleMeds was checked (crediblemeds.org) and if further information on frequency was still required, a search on PubMed was carried out. The information retrieved was examined and drugs were scored by two separate investigators (D.B. and S.G.). Where there was disagreement on the score, a discussion between the two investigators took place until agreement was achieved.

Drugs causing QTc prolongation were classified in Medichec as follows:

- QTc prolongation not reported in the BNF/drug not analysed for this side-effect.
- QTc prolongation has a conditional risk (caution/contraindication/interaction).
- QTc prolongation reported as rare or very rare.
- QTc prolongation reported as uncommon.
- QTc prolongation reported as common or very common.

The QTc prolongation side-effect was given an additional rating of conditional risk (Blue). This category included drugs associated with QTc prolongation or torsades de pointes but only under certain conditions of their use (e.g. excessive dose, in patients with conditions such as hypokalaemia, or when taken with interacting drugs). This includes drugs where QTc prolongation is listed in the BNF as a caution or contraindication, or under interactions, without it being listed as a side-effect as well. Because drug-induced QTc prolongation is relatively rare but can have serious consequences, it was deemed important to highlight conditional risks as well for this side-effect, and to carry out additional searches to identify frequency of its occurrence.

### Hyponatraemia

The methods described above were also used to identify drugs causing hyponatraemia. The search terms ‘hyponatraemia’ and ‘syndrome of inappropriate antidiuretic hormone’ or ‘SIADH’ were input in the NICE BNF database. Again, drug summaries that listed hyponatraemia in cautions, contraindications and drug interactions but not side-effects were also included. If the BNF listed the frequency as not known, the product information was checked for reported frequency and if further information was still required, a search on PubMed was carried out. Data retrieved were examined and drugs were scored by two separate investigators (D.B. and S.G.). Where there was disagreement, a discussion took place until agreement was achieved.

It was considered important for the side-effect hyponatraemia to have a further additional rating specific to some psychotropic agents considered less likely to cause hyponatraemia compared with those that are rated Amber. This category (Light amber) includes tricyclic antidepressants (TCAs) and some atypical antipsychotics. It is known that all antidepressants can cause hyponatraemia, but the BNF often lists this as ‘frequency not known’. Thus, where frequency could not be identified from either the BNF or product information, the Maudsley Prescribing Guidelines^45^ were consulted and, finally, a PubMed search was conducted. Evidence suggests that TCAs and other antidepressants are less likely to cause hyponatraemia than selective serotonin reuptake inhibitors (SSRIs) and serotonin and noradrenaline reuptake inhibitors (SNRIs),^45^ and they were therefore classed as Light amber so as to differentiate between the different classes of antidepressant. Those drugs that the BNF listed as having a frequency not known and only had case reports identified from PubMed were rated as Yellow.

For antipsychotic drugs, evidence suggests that first-generation antipsychotics (FGAs) are more likely to cause hyponatraemia than most second-generation antipsychotics (SGAs);^46^ however, even FGAs are less likely to cause hyponatraemia than antidepressants in general. Therefore, all FGAs were listed as Light amber. SGAs were listed as Light amber if the BNF stated that frequency was uncommon, rare or very rare. If frequency in the BNF was unknown, then a PubMed search was carried out and a decision was made on whether they were to be labelled Light amber or Yellow if only case reports were identified.

Drugs causing hyponatraemia were classified in Medichec as follows:

- Hyponatraemia not reported in the BNF/drug not analysed for this side-effect.
- Hyponatraemia has a conditional risk (caution/contraindication/interaction).
- Hyponatraemia case reports found only.
- Hyponatraemia reported as uncommon, rare or very rare but less likely to occur than drugs classed as Amber. This group comprises psychotropics only, including TCAs and some atypical antipsychotics.
- Hyponatraemia reported as uncommon, rare or very rare.
- Hyponatraemia reported as common or very common.

### Bleeding risk

For bleeding risk of drugs, the key terms used to search the BNF were ‘haemorrhage,’ ‘hemorrhage’, ‘bleed’, ‘blood loss’ and ‘haematuria’. Drugs were excluded if they were used to treat haemorrhages, as stated in the treatment summaries, or if they were given via an intramuscular route, since the route of administration itself, rather than the drug, can cause bleeding. The patient safety advice, cautions, contraindications and side-effect profiles in the BNF were analysed for the risk of haemorrhage. The side-effect profiles of the drugs were further scrutinised to differentiate the likely probability of the risk of haemorrhage occurring. Drugs listed with the side-effect of haemorrhage, but with the frequency unknown, were further cross-referenced using the product information on the emc website with the hope of securing further information on the likely probability of the risk of haemorrhage. Where information on frequency could not be ascertained, the drug was classed under conditional risk (Blue). This decision was taken on the basis that it was deemed important to highlight that bleeding could occur even if the frequency was not known and potentially very low, but owing to the severity of the side-effect it needed to be flagged. Furthermore, it was more difficult to identify case reports for drugs causing bleeding then for some of the other side-effects examined.

Drugs causing bleeding were classified in Medichec as follows:

- Bleeding not reported in the BNF/drug not analysed for this side-effect.
- Bleeding has a conditional risk (caution/contraindication/interaction), or frequency not known.
- Bleeding reported as rare or very rare.
- Bleeding reported as uncommon.
- Bleeding reported as common or very common.

### Constipation

Methods described above were used to identify drugs causing constipation using the keyword ‘constipation’ in the electronic BNF. Where frequency was not known in the BNF, the product information was checked on the emc website for the frequency with which constipation occurred. If no additional information was found, a PubMed literature search was carried out and all available evidence from published articles and case reports was used to assign a frequency after discussion between investigators (D.B. and S.R.). If frequency could still not be ascertained, the drug was classed as Grey, along with drugs that had not been analysed for this side-effect, since constipation is generally a common side-effect with many drugs and could be reported in the BNF in the future. This allows us to review this further down the line.

Drugs causing constipation were classified in Medichec as follows:

- Constipation not reported in the BNF/drug not analysed for this side-effect or frequency not known.
- Constipation has a conditional risk (caution/contraindication/interaction).
- Constipation reported as rare or very rare.
- Constipation reported as uncommon.
- Constipation reported as common or very common.

## Discussion

Medichec provides cogent, simple, portable and accessible information on adverse effects of medication. It can be accessed within a few seconds on a computer, smartphone or tablet in clinic or on home visits. Several versions exist incorporating regular updates. Having started as a resource primarily for memory assessment services to measure and communicate central anticholinergic burden, the Medichec website and app have been successively updated and developed to include additional side-effects that are paramount more widely to the safety of older people, those with intellectual disability and, potentially, other groups likely to receive polypharmacy. By having easily accessible side-effect information all in one place, this offers a relatively holistic support for prescribing, aiming to reduce human error and to prompt consideration and review when single or multiple agents are associated with potentially problematic side-effects. Through this support, it is hoped that a reduction might be achieved in the risk of falls and other serious adverse effects, including QTc prolongation, hyponatraemia, bleeding and cognitive impairment, in vulnerable adults. The app and web-based version are user friendly and can be used not only by GPs and doctors, but by pharmacists, other health professionals as well as patients and carers to help with optimising medication by reducing risk associated with side-effects, particularly when the person is taking several other medications. And although Medichec has been designed with psychiatrists and psychotropic prescribing in mind, clearly it may have applicability for other medical specialties and wider prescribing decisions.

Medichec is now being used across SLaM's older adult services, in other similar UK settings and globally. It is available online as a free resource at www.medichec.com and Android and Apple apps are also available. Plans are underway to see whether Medichec can be incorporated into GP health record systems to improve accessibility and safer prescribing for cognitively vulnerable patients in primary care. Finally, Medichec was selected for use in the Prescribing Observatory for Mental Health UK (POMH-UK) national audit on ‘Use of medicines with anticholinergic properties in older people's mental health services’ in summer 2023. Medichec version 3 (which will include the side-effects QTc prolongation, bleeding, hyponatraemia and constipation) will be available in autumn 2023.

### Limitations

Limitations of Medichec include the fact that the dose of medication is not considered when assessing the frequency or extent of the adverse effects. In addition, although we have tried to give a sense of the extent of the effect with some side-effects, such as central anticholinergic burden, this was not possible for all side-effects. Hyponatraemia may be more severe when several hyponatraemia-inducing drugs are taken together, and this should be considered when interpreting the results from Medichec. In addition, data on the extent of QTc prolongation were not available for all drugs so we rated drugs in terms of frequency of reports for QTc prolongation instead. This was supplemented with additional information where available, but it is important to note that one drug could cause QTc prolongation rarely but the effect could be clinically significant, whereas another drug could prolong QTc commonly but by a small effect of little clinical significance. Again, dose might play a role here as well and results should be interpreted carefully.

### Clinical implications

Medichec is a decision support tool that can facilitate access to information on various side-effects of multiple medications at once, aid clinical decision-making, optimise treatment and improve patient safety, especially in vulnerable adults.

## Data Availability

Data is available from the corresponding author, D.B., on reasonable request.

## About the authors

**Delia Bishara** is a member of the Mental Health for Older Adults and Dementia Clinical Academic Group, South London and Maudsley NHS Foundation Trust, London, UK; a researcher in the Department of Psychology at the Institute of Psychiatry, Psychology & Neuroscience, King's College London, London, UK; and consultant pharmacist for the mental health of older adults and dementia in the Pharmacy Department, South London and Maudsley NHS Foundation Trust, London, UK. **Sahar Riaz**, Institute of Psychiatry, Psychology & Neuroscience, King's College London, London, UK. **Justin Sauer**, Mental Health for Older Adults and Dementia Clinical Academic Group, South London and Maudsley NHS Foundation Trust, London, UK; and Institute of Psychiatry, Psychology & Neuroscience, King's College London, London, UK. **Christoph Mueller**, Mental Health for Older Adults and Dementia Clinical Academic Group, South London and Maudsley NHS Foundation Trust, London, UK; and Institute of Psychiatry, Psychology & Neuroscience, King's College London, London, UK. **Siobhan Gee**, Institute of Psychiatry, Psychology & Neuroscience, King's College London, London, UK; and Pharmacy Department, South London and Maudsley NHS Foundation Trust, London, UK. **David Taylor**, Institute of Psychiatry, Psychology & Neuroscience, King's College London, London, UK; and Pharmacy Department, South London and Maudsley NHS Foundation Trust, London, UK. **Robyn-Jenia Wilcha**, Mental Health for Older Adults and Dementia Clinical Academic Group, South London and Maudsley NHS Foundation Trust, London, UK. **Millie Edwards**, Mental Health for Older Adults and Dementia Clinical Academic Group, South London and Maudsley NHS Foundation Trust, London, UK. **Nirja Beehuspoteea**, Mental Health for Older Adults and Dementia Clinical Academic Group, South London and Maudsley NHS Foundation Trust, London, UK. **Anne Marie Bonnici Mallia**, Mental Health for Older Adults and Dementia Clinical Academic Group, South London and Maudsley NHS Foundation Trust, London, UK. **Jennifer Brook**, Mental Health for Older Adults and Dementia Clinical Academic Group, South London and Maudsley NHS Foundation Trust, London, UK. **Bharathi Balasundaram**, Department of Psychological Medicine, Changi General Hospital, Singapore. **Daniel Harwood**, Mental Health for Older Adults and Dementia Clinical Academic Group, South London and Maudsley NHS Foundation Trust, London, UK. **Nicola Funnell**, Mental Health for Older Adults and Dementia Clinical Academic Group, South London and Maudsley NHS Foundation Trust, London, UK. **Andre Strydom**, Institute of Psychiatry, Psychology & Neuroscience, King's College London, London, UK. **Robert Stewart**, Mental Health for Older Adults and Dementia Clinical Academic Group, South London and Maudsley NHS Foundation Trust, London, UK; and Institute of Psychiatry, Psychology & Neuroscience, King's College London, London, UK.
